# A combined computational and functional approach identifies IGF2BP2 as a driver of chemoresistance in a wide array of pre-clinical models of colorectal cancer

**DOI:** 10.1186/s12943-023-01787-x

**Published:** 2023-05-30

**Authors:** Sandra Kendzia, Susanne Franke, Tarek Kröhler, Nicole Golob-Schwarzl, Caroline Schweiger, Anna M. Toeglhofer, Christina Skofler, Stefan Uranitsch, Amin El-Heliebi, Julia Fuchs, Andreas Punschart, Philipp Stiegler, Marlen Keil, Jens Hoffmann, David Henderson, Hans Lehrach, Marie-Laure Yaspo, Christoph Reinhard, Reinhold Schäfer, Ulrich Keilholz, Christian Regenbrecht, Rudolf Schicho, Peter Fickert, Sigurd F. Lax, Frank Erdmann, Marcel H. Schulz, Alexandra K. Kiemer, Johannes Haybaeck, Sonja M. Kessler

**Affiliations:** 1 Institute of Pharmacy, Experimental Pharmacology for Natural Sciences, Martin Luther University Halle-Wittenberg, Halle, Germany; 2 Department of Pharmacy, Pharmaceutical Biology, Saarland University, Saarbrücken, Germany; 3 Diagnostic & Research Center for Molecular Biomedicine, Institute of Pathology, Medical University of Graz, Graz, Austria; 4 Center for Biomarker Research in Medicine (CBmed), Graz, Austria; 5 Department of Dermatology and Venereology, Medical University of Graz, Graz, Austria; 6 Charité Comprehensive Cancer Center, Charité - Universitätsmedizin Berlin, Berlin, Germany; 7 Department of Surgery, Hospital Brothers of Charity Graz, Graz, Austria; 8 Division of Cell Biology, Histology and Embryology, Gottfried Schatz Research Center, Medical University of Graz, Graz, Austria; 9 BioTechMed-Graz, Graz, Austria; 10 Division of Medical Physics and Biophysics, Medical University Graz, Graz, Austria; 11 Department of Surgery, Medical University of Graz, Graz, Austria; 12 Experimental Pharmacology & Oncology, Berlin GmbH-Berlin-Buch, Germany; 13 Bayer AG, Berlin, Germany; 14 Max Planck Institute for Molecular Genetics, Berlin, Germany; 15 Eli Lilly & Company, Indianapolis, USA; 16 CELLphenomics GmbH, Berlin, Germany; 17 Institute for Pathology, University Hospital Göttingen, Göttingen, Germany; 18 Division of Pharmacology, Medical University of Graz, Graz, Austria; 19 Division of Gastroenterology and Hepatology, Medical University of Graz, Graz, Austria; 20 Department of Pathology, Hospital Graz South-West and School of Medicine, Johannes Kepler University Linz, Linz, Austria; 21 Institute for Cardiovascular Regeneration, Goethe-University Hospital, Frankfurt, Germany; 22 Institute of Pathology, Neuropathology and Molecular Pathology, Medical University of Innsbruck, Innsbruck, Austria; 23 Halle Research Centre for Drug Therapy (HRCDT), Halle, Germany

**Keywords:** Drug resistance, Neoplasm, Colorectal neoplasms, RNA-binding proteins

## Abstract

**Aim:**

Chemoresistance is a major cause of treatment failure in colorectal cancer (CRC) therapy. In this study, the impact of the IGF2BP family of RNA-binding proteins on CRC chemoresistance was investigated using in silico, in vitro, and in vivo approaches.

**Methods:**

Gene expression data from a well-characterized cohort and publicly available cross-linking immunoprecipitation sequencing (CLIP-Seq) data were collected. Resistance to chemotherapeutics was assessed in patient-derived xenografts (PDXs) and patient-derived organoids (PDOs). Functional studies were performed in 2D and 3D cell culture models, including proliferation, spheroid growth, and mitochondrial respiration analyses.

**Results:**

We identified IGF2BP2 as the most abundant IGF2BP in primary and metastastatic CRC, correlating with tumor stage in patient samples and tumor growth in PDXs. IGF2BP2 expression in primary tumor tissue was significantly associated with resistance to selumetinib, gefitinib, and regorafenib in PDOs and to 5-fluorouracil and oxaliplatin in PDX in vivo. *IGF2BP2* knockout (KO) HCT116 cells were more susceptible to regorafenib in 2D and to oxaliplatin, selumitinib, and nintedanib in 3D cell culture. Further, a bioinformatic analysis using CLIP data suggested stabilization of target transcripts in primary and metastatic tumors. Measurement of oxygen consumption rate (OCR) and extracellular acidification rate (ECAR) revealed a decreased basal OCR and an increase in glycolytic ATP production rate in *IGF2BP2* KO. In addition, real-time reverse transcriptase polymerase chain reaction (qPCR) analysis confirmed decreased expression of genes of the respiratory chain complex I, complex IV, and the outer mitochondrial membrane in *IGF2BP2* KO cells.

**Conclusions:**

IGF2BP2 correlates with CRC tumor growth in vivo and promotes chemoresistance by altering mitochondrial respiratory chain metabolism. As a druggable target, IGF2BP2 could be used in future CRC therapy to overcome CRC chemoresistance.

**Supplementary Information:**

The online version contains supplementary material available at 10.1186/s12943-023-01787-x.

## Introduction

Colorectal cancer (CRC) is the third most common malignancy and the second most common cause of cancer related deaths worldwide [1]. The 5-year survival rate differs greatly, from 90% in early stages to 14% in advanced stages [2].

The current common treatment approach includes surgery, chemotherapy, and radiotherapy [3]. In the early stages of CRC, the main therapy remains surgery. Adjuvant chemotherapy is indicated particularly in advanced stages to reduce the risk of recurrence. Only a subset of advanced CRCs responds to the most commonly used chemotherapeutics, 5-fluorouracil, oxaliplatin, or irinotecan [4]. This is primarily due to chemoresistance, which can lead to chemotherapy failure and subsequent recurrence and is hence a major clinical issue [3, 5, 6]. Different causes and mechanisms have been postulated for chemoresistance in CRC, e.g. via signaling pathways like NFKB, Wnt/ β-catenin, and PI3K/AKT which lead to ABC transporters overexpression or the overexpression of Thymidylate synthase and FOXO1 proteins in CRC [3, 6]. Predictive biomarkers for chemoresistance in CRC are still lacking. BRAF, KRAS and NRAS mutations are routinely used as biomarkers for a resistance against anti- epidermal growth factor receptor (EGFR) therapy [7]. Nonetheless novel biomarkers are needed, since EGFR treatment with cetuximab is not sufficient in a significant part of wild type tumors [7, 8]. Thus, prognosis in non-metastatic CRC cannot be adequately described by these biomarkers. Further, there is still a need for more effective second-line therapies in advanced CRC. Therefore, new therapeutic targets and an insight into the mechanisms of chemoresistance are needed to improve CRC patients´ outcome.

The insulin-like growth factor 2 (IGF2) mRNA binding protein (IGF2BP/IMP/VICKZ) family has been reported to regulate subcellular mRNA localization, stability, and translation [9]. IGF2BP3 is known to support the development of chemoresistance in breast cancer cells [10], whereas IGF2BP1 was shown to promote chemoresistance in ovarian cancer [11, 12], rhabdomyosarcoma [13], and melanoma [14]. For IGF2BP2, its splice variant p62 has been shown to influence chemoresistance due to antiapoptotic actions [15] and a more aggressive tumor phenotype [16] in hepatocellular carcinoma (HCC). IGF2BP2 is overexpressed in CRC and has been reported to foster cell proliferation, to increase cell cycle progression, and to inhibit early apoptosis [17, 18]. Furthermore, IGF2BP2 is linked to tumor growth and poor outcome in other cancer types, such as HCC [15, 19], gallbladder cancer [20], pancreatic cancer [21], and glioblastoma [22–24]. IGF2BP2 is able to activate the phosphoinositide 3-kinase/protein kinase B (PI3K/AKT) pathway at least in part by binding to *IGF2* mRNA and downstream activation of the insulin-like growth factor 1 receptor (IGF1R) [25, 26]. Well known inhibitors of the PI3K-AKT pathway, phosphatase and tensin homolog (PTEN) and insulin-like growth factor 2 receptor (IGF2R) are able to counteract IGF2BP2-induced PI3K activation [27]. In light of these data, IGF2BP2 seems to be a promising target to influence chemoresistance in CRC.

Additionally, IGF2BP2 has been shown to influence the oxidative phosphorylation (OXPHOS) in glioblastoma cancer stem cells [28]. Similarly, it is reported, that IGF2BP2 disrupts the oxidative metabolism in cardiomyocytes [29] and affects mitochondrial function in the heart [30]. In general, metabolic rewiring is typical for cancer cells. A better understanding of the influence of IFG2BP2 on the metabolism of CRC may lead to additional therapy strategies targeting chemoresistance in CRC.

The aim of this study was to investigate the role of the IGF2BPs in CRC and their potential impact on chemoresistance. In order to do so, we made use of a well characterized patient cohort [31] in combination with patient derived xenograft (PDX) and patient derived organoid (PDO) models as well as functional approaches in 2D and 3D cell culture.

## Methods

### RNA-Seq data

RNA-Seq data from primary and metastatic CRC samples, PDOs, and PDXs were used as available from the previous study [31]. This included, for primary CRC samples processed expression data from 88 tumor tissues and 22 normal liver tissues. For metastatic CRC samples data from 23 tumor tissues and 12 normal liver tissues. RNA-seq expression counts had been normalized as reads per kilobase of transcript per million mapped reads (RPKM). RPKM values were used for further analyses as done in the original publication. RPKM values of genes of interest were tested for normal distribution and then an either parametric or non-parametric test was used to test for differential expression between groups or genes within the same tissue/organ.

### Tumor tissue

Tumor material was received with informed consent from 40 CRC patients from the St. John of God Hospital and the University Hospital Graz together with clinical information under approval by the ethics committees of the Medical University Graz and St. John of God Hospital (23–15 ex 1/11) [32]. Pathological processing was performed at the Department of Pathology, Hospital Graz II and the Diagnostic & Research Institute of Pathology of the Medical University Graz and pathological data were provided. All samples used for IHC analysis were also part of the patient cohort published by Schütte et al. [31].

### Analyses of PDX and PDO data

Animal experiments as well as organoid culture was performed in the original study [31]. Data on tumor growth of PDX under the respective treatments or vehicle control were used for correlation analyses. For analysis of the resistance data of the PDO samples, log10 IC50 values, which can be found in Supplementary Data 14 of Schütte et al. [31], were used.

### Immunohistochemistry (IHC)

IHC was performed on a Ventana Immunostainer XT (Ventana Medical Systems, Tucson, USA), using an ultra-VIEW universal DAB Detection Kit (Ventana Medical Systems, Tucson, USA) and cell conditioning solution for 30 min using heat induced epitope retrieval. The primary customized IGF2BP2/IMP2/p62 antibody [33, 34] was incubated for 30 min using a dilution of 1:1000.

Two observers (N. GS., J. H.), blinded to the clinical data related to the respective cases, evaluated all stains independently on a light microscope. IGF2BP2 expression was evaluated with respect to staining intensity (intensity score 0–3; 0 no staining, 1 weak, 2 moderate and 3 strong) and percentage of positive cells (proportion score; 0–100%). In discrepant cases, the average score was taken into account. The same scoring strategy has been used in previous published studies [35, 36].

### Cell culture

HCT116, which are human colorectal carcinoma cells, were maintained in Dulbecco’s modified Eagle’s medium (DMEM, #21969-035, gibco). Medium was supplemented with 10% fetal calf serum (FCS, #P30-3306, PAN-Biotech), 1 mM glutamine (#X0551-100, Biowest), 100 U/mL penicillin, and 100 μg/mL streptomycin (#15070-063, Gibco). The cells were cultured at 37 °C and 5% CO2. Cell line authentication was conducted by STR/DNA profiling. For cell line authentification the Cell line Authentification Service by Eurofins Genomics was used. DNA isolation was carried out from cell pellet. Genetic characteristics were determined by PCR-single-locus-technology. 16 independent STR loci D8S1179, D21S11, D7S820, CSF1PO, D3S1358, TH01, D13S317, D16S539, D2S1338, AMEL, D5S818, FGA, D19S433, vWA, TPOX, and D18S51 were investigated using the AmpFlSTR® Identifiler® Plus PCR Amplification Kit (Thermo Fisher). In parallel, positive and negative controls were carried out yielding correct results. Results were compared to a reference sample and known profiles. Mycoplasma testing was performed regularly via PCR. For that purpose, cells were grown to confluence over several days in media without antibiotics. A PCR was performed with the supernatant of the cells (Primer forward 5´-3´: GGCGAATGGGTGAGTAACACG, Primer reward 5´-3´: CGGATAACGCTTGCGACCTATG). Positive samples reveal a distinct 500 bp band on the agarose gel.

CRISPR-mediated IGF2BP2 knockout cells have been previously published: all experiments in the current study were performed with cell clone KO#1 from the previous study [17]. Confirmation of *IGF2BP2* KO was confirmed on a regular basis by Western blot analysis (Supplementary methods, Supplementary fig. S1). The overexpression plasmid and the control vector were previously published [15] and are deposited with Addgene (www.addgene.org; plasmid 42175, plasmid 42174). HCT116 cells were transfected with Lipofectamin 3000 (#L3000015, Thermo Fischer) according manufacturer instructions using 0.5 µl Lipofectamin 3000 and 500 ng plasmid DNA per well of a 24-well plate. For OCR/ECAR measurements cells were transfected 24 h post seeding to a 24-well plate. After two days of cultivation, cells were trypsinized and seeded to Seahorse XF96 V3 PS Cell Culture Microplates (#101085-004, Agilent) and experiments were conducted as described below.

### 2D and 3D Proliferation

The cells were treated with 5-fluorouracil (22.2 µM, stock 40 mM in DMSO, #F6627, Sigma Aldrich), oxaliplatin (2.6 µM, stock 4 mM in PBS, #Y0000271, European Directorate of the Quality of Medicines & HealthCare), gefitinib (23.5 µM, stock 50 mM in DMSO, #G-4408, LC Laboratories), regorafenib (8.1 µM, stock 50 mM in DMSO, #R-8024, LC Laboratories), selumitinib (7.4 µM, stock 50 mM in DMSO, #S-4490, LC Laboratories) or nintedanib (8.2 µM, stock 25 mM in DMSO, #N-9077, LC Laboratories). Stock solutions were stored in aliquots at -20 °C and used within one month after preparation. The concentrations used were determined as IC50 values for HCT116 wildtype by a resazurin assay. For this viability assay, cells were seeded and 24 h later treated with a concentration spectrum of the respective compound. After 48 h of treatment, cells were incubated with resazurin (0.02 mg/ml, stock 10× in PBS, #R7017, Sigma Aldrich) for 4 h. Detection was performed as previously described [37].

For the 2D kinetic proliferation analysis, 5000 cells were seeded per well into 96-well plates. The next day, cells were treated with the respective compounds, and cell confluency was monitored in an IncuCyte® S3 system for a period of 4 days. Cell confluency was analyzed using IncuCyte® basic analyzer software. The confluency was normalized to the time of treatment. The normalized values were substracted from the proper vehicle control.

For the 3D proliferation analysis, 3000 cells were seeded per well into low-attachment U-bottom 96-well plates. After spheroid formation for 3 days, spheroids were treated with the respective compounds, and monitoring in an IncuCyte® S3 system was started. The spheroid area was analyzed using the spheroid IncuCyte® software, and the area was normalized to the first measuring time point after treatment (0 h).

### CLIP data analysis and gProfiler enrichment analysis

Publicly available enhanced CLIP (eCLIP) RNA-binding data from the ENCODE consortium for HepG2 liver cancer cells and K562 cells was used [38]. eCLIP peaks were obtained from the ENCODE data portal (https://www.encodeproject.org/) and analyzed as previously published [39]. Each annotated human gene in the Ensembl database, that had an eCLIP peak in at least one of the two cell lines, was denoted as an IGF2BP2 target gene (IGF2BP +). We used a Kolmogorov-Smirnow test on the distribution of all genes and IGF2BP + or IGF2BP- genes to compute significant changes in log fold changes using R. In order to obtain genes that are differentially expressed between normal and cancer or metastastic samples, we used the Mann-Whitney U Test. Resulting gene *p*-values were corrected for multiple testing using an FDR-based approach [40]. We used all differentially expressed target genes that had a log fold change of > 0.5 for the gene set enrichment analysis with g:Profiler [41].

### OCR/ECAR measurement

The Mito Stress Test and the ATP Rate Assay were performed using an Agilent Seahorse 96XF device and respective kits. The assays were performed as described in the manufacturer’s protocol (#103592-100, #103015-100, Agilent). In brief, the cells were seeded 24 h before measurement. The medium was replaced one hour prior to measuring by seahorse XF DMEM assay medium (#103575-100, Agilent) supplemented by 10 mM glucose (#103577-100, Agilent), 1 mM sodium pyruvate (#103578-100, Agilent) and 2 mM glutamine (#103579-100, Agilent). For the Mito Stress Test, cells were treated with 2 μM oligomycin, 0.5 μM FCPP, 0.5 μM rotenone/antimycin A (Seahorse XF Cell Mito Stress Test Kit, #103015-100, Agilent) and 4 µM Hoechst (#62249, Thermo Scientific). The ATP Rate Assay included adding of 2 μM oligomycin followed by 0.5 μM rotenone/antimycin A and Hoechst to the cells. The data were analyzed by the Seahorse Wave Software (Agilent Technologies, Santa Clara, CA, USA). After measurements with the Seahorse XF Analyzer, cells were counted using the Bio Tek Cytation 5 Cell Imaging Multimode Reader for normalization by Hoechst staining.

### qPCR

Total RNA was isolated using the High Pure RNA Isolation Kit (#11828665001, Roche). Concentration of isolated RNA was quantified by NanoDrop (Thermo Fisher Scientific), and RNA with an A260/A280 ratio higher than 1.7 was used for further experiments. RNA was reverse transcribed using the High-Capacity cDNA Reverse Transcription Kit (#4368813, Thermo Fisher Scientific) in the presence of an RNase inhibitor (#10777-019, Invitrogen) according to the manufacturer’s instructions. cDNA was analyzed using 5 × HotFirePol EvaGreen qPCR Mix (#08-25-00020, Solis BioDyne) with a CFX96 real-time PCR system (Bio-Rad). Primer sequences can be found in Supplementary table S1. qPCR data were analyzed with the Bio-Rad CFX Maestro 1.1 Software 2017 (Bio-Rad Laboratories). Reference genes were discriminated based on the GeNorm algorithm [42] which is included in the Maestro software. *18S* was found to be more stable in its expression pattern compared to *ACTB* due to a lower M value (internal control gene-stability measure, M < 0.5)*.* Consequently, data were normalized to *18S* and are shown relative to the control.

### Statistical analysis

Data analysis and statistics were performed using OriginPro. IC50 values were calculated using sigmoidal fitting with Origin pro version 19 software. Data are represented as means ± SEM if not indicated otherwise. Depending on whether the data were normally distributed and on the group size, statistical differences were calculated using one-way or two-way ANOVA, Student’s t-test, or Mann-Whitney *U* Test. Linear correlation was measured with the Pearson correlation coefficient or Spearman´s rank correlation coefficient. Fisher´s exact test was used in the analysis of contingency tables. #/*p < 0.05; **p < 0.01; ***p < 0.001. Biological replicates were represented as “*n*” and technical replicates were represented in brackets.

## Results

The RPKM values of the three IGF2BP family members were analyzed using RNA-Seq data of the published patient cohort by Schütte and colleagues [31].

Schütte et al. established a biobank of a patient cohort with 106 CRC tumors (stages I-IV), 35 patient derived organoids (PDO) and 59 patient derived xenografts (PDX) with extensive molecular characterization. In the original study was the tumor genomic and transcriptomic landscapes with the derived pre-clinical models compared by integrating whole genome (WGS), whole exome (WES) and RNA sequencing data. In addition, from a part of the cohort FFPE tissues were available. The vast majority of patients of the cohort were therapy-naïve.

**Fig. 1:**
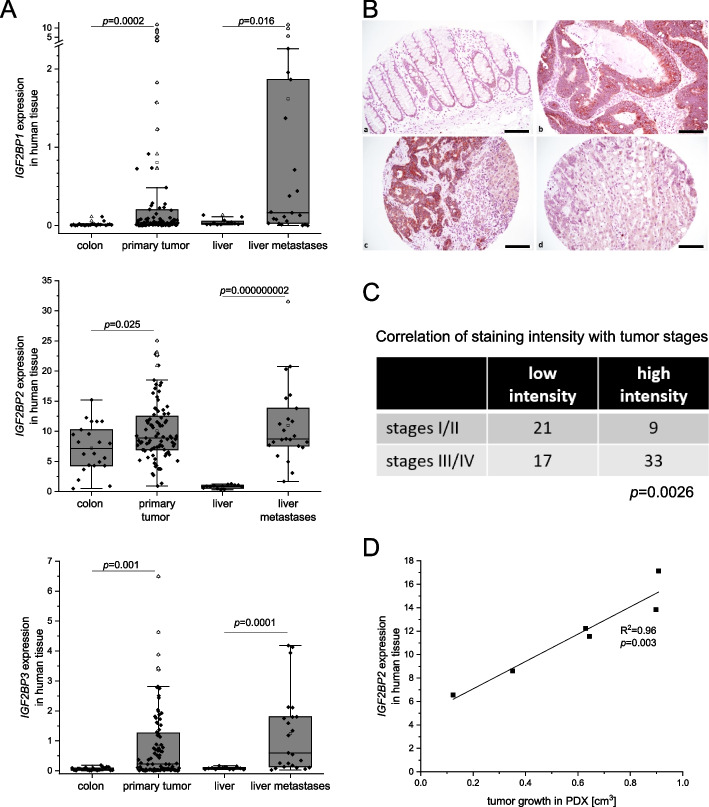
IGF2BP2 is associated with tumor growth and patients’ outcome in CRC. **A** RNA expression using RPKM values of *IGF2BP1-3* in normal colon tissue (colon, *n* = 22), primary tumor tissue (primary tumor, *n* = 88), non-neoplastic hepatic tissue (liver, *n* = 12), and tissue from liver metastases (liver metastases, *n* = 23) was determined by RNA-Seq [31]. Statistical significance was determined by Mann-Whitney *U* Test as data were not normally distributed. **B** Representative images of immunohistochemical staining against IGF2BP2 in a) normal colon tissue, b) primary tumor tissue, c) and d) liver metastasis with normal hepatic tissue. Scale bar = 100 µm, original magnification was 200x. **C** Immunohistochemistry for IGF2BP2 was scored for staining intensity. Samples with low (scores 1–2.49) or high intensity (score 2.5–3) were grouped regarding tumor stages I/II and III/IV. Statistical significance was determined by Fisher´s exact test. **D** Tumor growth in cm^3^ of PDXs was correlated with *IGF2BP2* expression of primary tumor tissue. Pearson correlation is shown for day 46 after tumor transplantation

All three *IGF2BP*s were overexpressed on RNA level in both primary tumors and hepatic metastases with *IGF2BP2* being the most abundant IGF2BP family member compared to *IGF2BP1* and *IGF2BP3* due to higher basal expression levels (*n* = 74; Fig. 1A, Supplementary table S2). In order to confirm IGF2BP2 overexpression on protein level, immunohistochemistry was performed on paraffin sections from the same patient cohort. Interestingly, a higher IGF2BP2 immunohistochemical staining intensity was associated with more advanced tumor stages in tissue microarrays (*n* = 80; Fig. 1B, C). Concordantly, expression of *IGF2BP2* significantly correlated with tumor growth in PDX models in the late tumor growth phase 46 days after tumor transplantation (Fig. 1D).

**Fig. 2:**
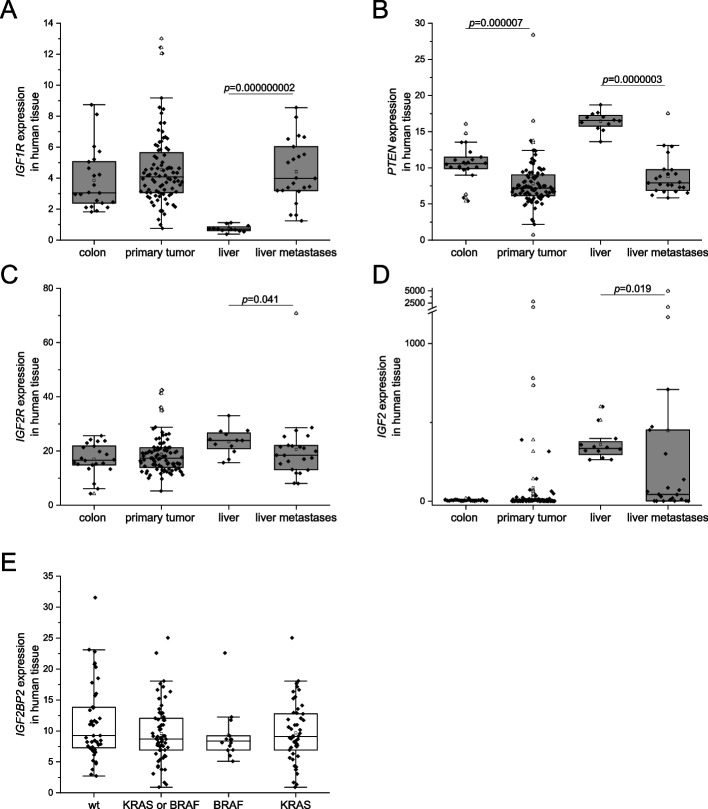
RNA expression of members of the *IGF2/AKT* axis in human CRC samples and independency of IGF2BP2 expression regarding *BRAF* and* KRAS* mutational status. **A**-**D** RNA expression using RPKM values of *IGF2*, *IGF1R*, *PTEN*, and *IGF2R* from RNA-Seq data [31] of primary tumor tissue (primary tumor, *n* = 88), normal colon tissue (colon, *n* = 22), tissue from liver metastases (liver metastases, *n* = 23), and non-neoplastic hepatic tissue (liver, *n* = 12). **E** IGF2BP2 expression in primary and metastatic tumors grouped in *BRAF* and *KRAS* wild-type (wt) samples, samples carrying either *KRAS* or *BRAF* mutations (*KRAS* or *BRAF*), and samples with *BRAF* (*BRAF*) or *KRAS* (*KRAS*) mutations. Statistical significance was determined by Mann-Whitney *U* Test as data were not normally distributed

RNA-Seq data [31] further revealed that the downstream target of the IGF2BP2-IGF2 axis, *IGF1R*, was significantly increased in metastatic tissues (Fig. 2A), whereas the inhibitor of the PI3K-AKT pathway *PTEN* was decreased in both primary and metastatic tumor tissue (Fig. 2B). Concordantly, the tumor suppressor *IGF2R* was decreased in metastatic tissues (Fig. 2C). *IGF2* expression on the other hand, was not altered in primary tumor tissue, but decreased in metastatic tumor tissue (Fig. 2D). Interestingly, *IGF2BP2* expression was independent of *KRAS* or *BRAF* mutations (Fig. 2E).

**Fig. 3:**
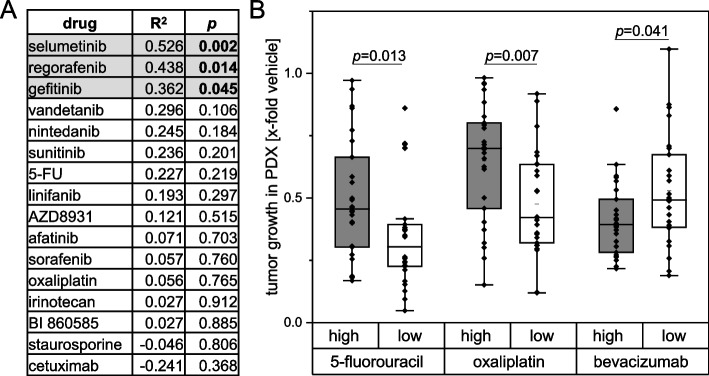
*IGF2BP2* expression is associated with chemoresistance in PDOs and *in* PDXs. **A** Table shows Pearson correlation coefficients (R^2^) and respective *p*-values for correlation analysis of *IGF2BP2* expression of primary tumor tissues with logIC50 values of the respective drug in the matched PDOs. IC50 values have previously been published [31]. Significant correlations are depicted by grey background color. **B** PDX samples of primary tumor and liver metastasis tissue (*n* = 52) were divided in high (above median) and low (below median) *IGF2BP2* expressing groups. PDX tumor growth under treatment with the respective drug was plotted. Statistical significance was determined by Mann-Whitney *U* Test for not normally distributed samples or Student´s t-test for normally distributed samples

Since the PI3K-AKT pathway was described as a key link to chemoresistance [43], we hypothesized that IGF2BP2 might lead to chemoresistance in CRC. Thus, resistance data from PDOs was correlated with *IGF2BP2* expression. The correlation of log IC50 values in PDOs with matched *IGF2BP2* expression in the primary tumor of the respective patient was analyzed (for details on PDOs and patient data see [31]). *IGF2BP2* expression of primary tumor tissue significantly correlated with resistance against selumetinib, gefitinib, and regorafenib in PDOs (Fig. 3A, Supplementary fig. S2). Furthermore, *IGF2BP2* expression correlated with chemoresistance in PDXs treated with the respective chemotherapeutic compound. PDX samples were divided by the median into high *IGF2BP2* and low *IGF2BP2* expressing samples. High *IGF2BP2* expressing tumors were less responsive against 5-fluorouracil and oxaliplatin (Fig. 3B, Supplementary fig. S3). However, bevacizumab treatment was slightly less effective in *IGF2BP2* low expressing tumors (Fig. 3B). Concordantly, Spearman correlation coefficients of *IGF2BP2* expression with PDX tumor growth for the latter drugs was *R*^*2*^ = 0.31, *p* = 0.02 for oxaliplatin, *R*^*2*^ = 0.30, *p* = 0.03 for 5-fluorouracil, and *R*^*2*^ = -0.34, *p* = 0.01 for bevacizumab.

**Fig. 4:**
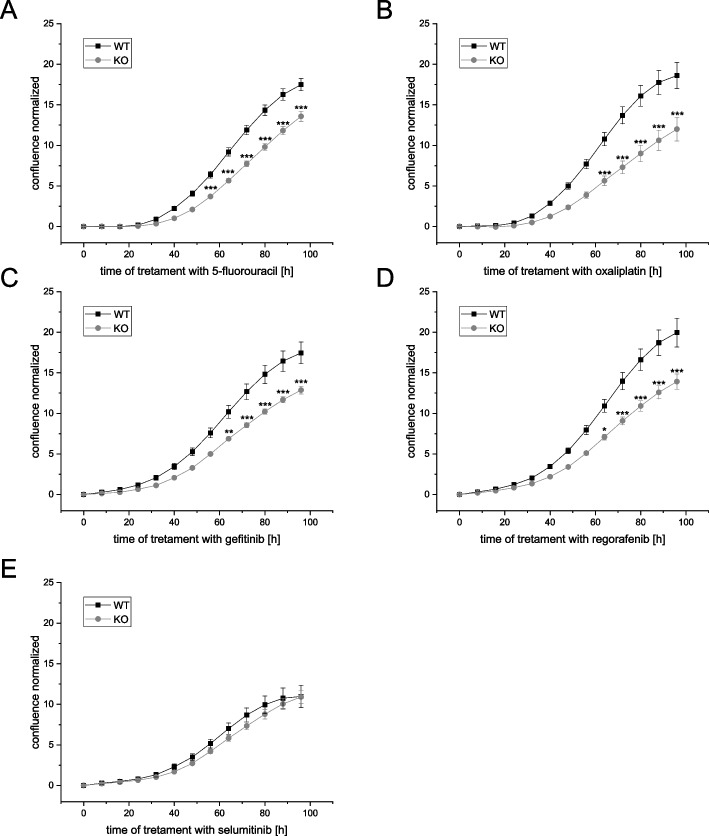
Chemoresistance of HCT116 IGF2BP2 wildtype (WT) and knockout (KO) cells in 2D cell culture. **A**-**E** Cell confluency was monitored using the IncuCyte®S3 system over 4 days. HCT116 WT cells and HCT116 *IGF2BP2* KO cells were seeded 24 h prior treatment. The added compounds were 5-fluorouracil (**A**, 22.2 µM), oxaliplatin (**B**, 2.6 µM), gefitinib (**C**, 23.5 µM), regorafenib (**D**, 8.1 µM) and selumitinib (**E**, 7.4 µM). Confluency was normalized to the time of treatment (0 h). Each normalized value was subtracted from its vehicle control. Data are represented as means ± SEM, *n* = 3 (quadruplicates). Statistical significance was tested by one-way ANOVA. *p ≤ 0.05, **p ≤ 0.01, ***p ≤ 0.001

Thus, HCT116 *IGF2BP2* knockout (KO) cells and HCT116 wildtype (WT) cells were tested for their responsiveness to these drugs. 10 different concentrations were used to determine IC20 and IC50 values (data not shown). KO cells were tested compared to WT cells using IC50 and IC20 of the respective drug. Indeed, *IGF2BP2* KO cells were more susceptible to IC50 of 5-fluorouracil, oxaliplatin, gefitinib, and regorafenib compared to parental wildtype cells (Fig. 4), which mostly recapitulated the growth inhibitory effect of the *IGF2BP2* KO (Supplementary figs. S4 and S5) as previously published [17]. Still, *IGF2BP2* KO cells were more susceptible to regorafenib treatment when treated with the IC20 concentration (Supplementary fig. S5). The assay with nintedanib completely restrained cell growth in 2D cell culture (data not shown).

**Fig. 5:**
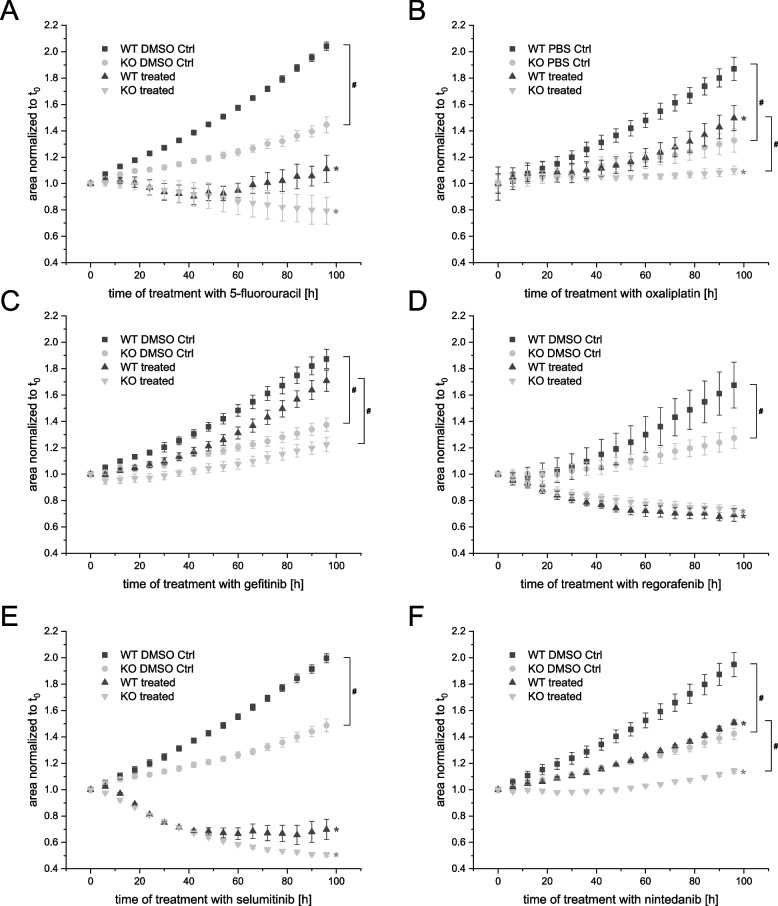
Chemoresistance of HCT116 *IGF2BP2* wildtype (WT) and knockout (KO) cells in 3D cell culture. **A**-**F** Spheroid growth of HCT116 WT and HCT116 *IGF2BP2* KO cells was monitored for 96 h by automated live-cell microscopy, starting after spheroid formation. Cells were treated with IC50 concentrations of 5-fluorouracil (**A**, 22.2 µM), oxaliplatin (**B**, 2.6 µM), gefitinib (**C**, 23.5 µM), regorafenib (**D**, 8.1 µM), selumitinib (**E**, 7.4 µM), nintedanib (**F**, 8.2 µM) and the vehicle control (Ctrl). Spheroid area was analyzed using the IncuCyte®S3 system and was normalized to 3-day old spheroids. Data are presented as means ± SEM, *n* = 3 (quadruplicates). Statistical analysis was performed with a two-way ANOVA using the area under the curve. Asterisks represent *p* values for the comparisons between the untreated and treated condition in the respective cell line. Hashmarks stand for *p* values labeling differences in growth between both cell lines either control or treated condition, *p* values were *p* ≤ 0.05

In 3D cell culture, which better recapitulates in vivo tumor growth, a growth inhibiting effect of *IGF2BP2* KO was confirmed. Beside this basal difference of *IGF2BP2* KO compared to WT cells as previously published [17], *IGF2BP2* KO cells were more susceptible against oxaliplatin and nintedanib compared to WT spheroids. Treatment efficiency of 5-fluorouracil, regorafenib, and gefitinib was equal in WT and KO cells (Fig. 5). Interestingly, using IC20 concentrations, *IGF2BP2* KO cells were also more susceptible against selumetinib (Supplementary fig. S6).

**Fig. 6:**
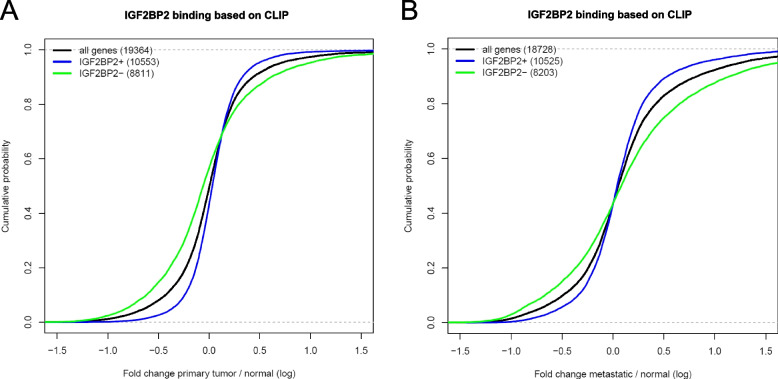
Analysis of IGF2BP2 targets in tumor and metastatic CRC tissues. **A**, **B** Cumulative plot of gene expression (log2 fold change) in primary (**A**) and metastatic (**B**) CRC tissues compared to their respective control tissues. Gene subgroups were built according to IGF2BP2 binding determined by IGF2BP2 CLIP data (see Methods) and representing all genes (black), IGF2BP2 positive genes (IGF2BP2+ ,blue) and IGF2BP2 negative genes (IGF2BP2-,green). Numbers in brackets denote the number of genes in each category

In order to decipher the mechanism of IGF2BP2-induced chemoresistance in CRC we integrated available IGF2BP2-CLIP data. It has been suggested that IGF2BP2 binds to target mRNAs and leads to increased stabilization [39, 44–46]. We predicted target mRNAs of IGF2BP2 using publicly available IGF2BP2 CLIP data from human HepG2 and K562 cells. Then we asked if there is a change in expression of IGF2BP2 targets in tumor versus normal tissues. Therefore, genes were dissected into groups with or without IGF2BP2 binding, and the total of all expressed genes. Figure 6A shows that genes bound by IGF2BP2 (IGF2BP2+) show significantly higher expression in tumor tissues (Kolmogorov-Smirnow test, *p* < 2.2e-16). Non-target genes (IGF2BP2-) showed lower fold changes than the gene average. Similar results could be found in metastatic versus normal liver tissues (Fig. 6B; Kolmogorov-Smirnow test, *p* < 2.2e-16). These analyses might suggest that overexpression of IGF2BP2 in CRC stabilizes IGF2BP2 target transcripts.

**Fig. 7:**
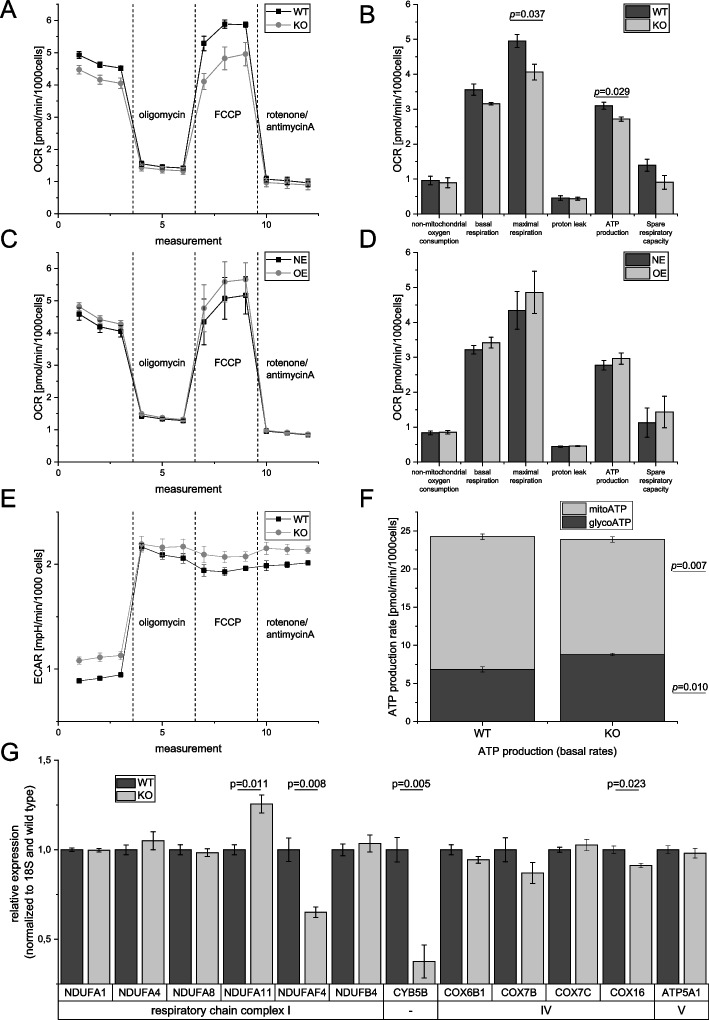
Bioenergetic profile in cells with modulated IGF2BP2 expression. Oxygen consumption rate (OCR) and extracellular acidification rate (ECAR) were measured using a Seahorse 96XF Analyzer in HCT116 *IGF2BP2* wildtype (WT) and knockout (KO) cells (**A**, **B**, **E**, **F**) or in HCT116 cells under control conditions (NE) or overexpressing IGF2BP2 (OE) (**C**, **D**). **A**-**E**: A Seahorse XF Cell Mito Stress Test was performed. After measuring basal OCAR and ECAR oligomycin was injected to shut down OXPHOS-dependent ATP production followed by adding FCCP as an encoupler to obtain the maximal mitochondrial respiration capacity. Rotenone/antimycine A shut down mitochondrial oxygen consumption by inhibiting respiratory chain complex I and III. Statistical analysis was performed using Student´s t-test. Data are shown as means ± SEM, *n* = 3 (4–8). **F** The Seahorse XF Real-Time ATP Rate Assay was conducted using the same concentration of oligomycin and rotenone/antimycine A (see methods). Statistical analysis was performed using Student´s t-test. Data are shown as means ± SEM; *n* = 3–4 (4–8). **G** Gene expression of the respiratory chain complex genes was performed by real-time RT-PCR analysis in HCT116 WT and HCT116 *IGF2BP2* KO cells. Gene expression data were normalized to the housekeeping gene 18S. Data are shown as means ± SEM relative to the WT control; *n* = 3 (triplicates). Statistical analysis was performed using Student´s t-test

A GSEA analysis using gProfiler [41] of all IGF2BP2 targets that are differentially expressed and showing a log2 fold change > 0.5 in tumor versus normal and/or metastastatic versus normal liver tissues revealed an enrichment being most significant for Gene Ontology (GO) biological processes and REACTOME pathways linked to cell cycle (Supplementary tables S3, S4 and S5). Interestingly, cell cycle proteins have been shown to directly upregulate mitochondrial respiration [47].OXPHOS has been shown to characterize chemoresistant cells in different cancer types and can be mediated by IGF2BP2, which has been shown to link IGF2BP2 expression with chemoresistance in glioblastoma [28]. To detect an effect on the aerobic and anaerobic metabolism, the OCR as an indicator for OXPHOS and the ECAR were determined using a Seahorse 96XF Analyzer. Concordantly, HCT116 IGF2BP2 KO cells showed a reduced basal OCR. Maximal respiration as well as ATP production were significantly reduced revealed by a Seahorse XF Cell Mito Stress Test (Fig. 7A, B). Vice versa, IGF2BP2 overexpression tended towards a slight increase in OCR of maximal respiration (Fig. 7C, D). Basal ECAR also changed after IGF2BP2 modulation suggesting an impact on glycolysis (Fig. 7E). The Seahorse XF Real-Time ATP Rate assay revealed a metabolic switch by observing an increase in glycolytic ATP Production Rate and a decrease in mitochondrial ATP Production Rate of IGF2BP2 KO cells while the total ATP Production Rate did not differ compared to WT cells (Fig. 7F). However, mitochondrial ATP Production Rate was not altered in HCT116 cells overexpressing IGF2BP2 (data not shown). To investigate the effects of *IGF2BP2* knockout on gene expression of some genes involved in the respiratory chain, they were analyzed by qPCR. The selection of genes was made as follows: the top three candidates from the CLIP analysis, i.e. *COX7C*, *ATP5A1*, *NDUFA4*, all of which showed peaks in all published CLIP datasets, as well as candidates known from the literature [28, 29]. qPCR analysis confirmed decreased expression of the respiratory chain complex I gene NADH:ubiquinone oxidoreductase complex assembly factor 4 (*NDUFAF4),* the outer mitochondrial membrane cytochrome B5 type B (*CYB5B*), and complex IV gene cytochrome C oxidase assembly factor COX16 (*COX16)* in HCT116 *IGF2BP2* KO cells. Interestingly, NADH:ubiquinone oxidoreductase subunit A11 (*NDUFA11)* was significantly upregulated in IGF2BP2 KO cells (Fig. 7G).

## Discussion

In this study, we used an integrated approach employing clinical data, PDOs and PDXs as well as 2D and 3D cell culture models in order to shed light on whether and how IGF2BP2 drives chemoresistance in CRC. We can demonstrate here for the first time, that IGF2BP2 indeed correlates with tumor growth and chemoresistance in vivo. Furthermore, causation was detected in cell lines by genetic perturbation of IGF2BP2.

A decade ago, autoantibodies against IGF2BP2 were detected in the sera of colon cancer patients [48]. Meanwhile, IGF2BP2 has been reported to promote CRC cell proliferation via different mechanisms [18, 49]. Further, it has been linked to poor prognosis in CRC patients [17]. However, data on the impact of IGF2BP2 on chemoresistance in colon cancer has been missing so far. Thus, our study has utilized a well characterized patient cohort [31] to be able to investigate the effect of IGF2BP2 on drug-specific chemoresistance in vitro and in vivo. Finally, a mechanism causing IGF2BP2-induced chemoresistance was postulated.

IGF2BP2 is known from the literature to be overexpressed in CRC tissues [18, 49], which could be confirmed by our study. Further, other factors of the PI3K/AKT pathway were differentially expressed in primary and metastatic tumor tissues. The link to members of the PI3K pathway is known in other non-neoplastic as well as malignant tissues [26, 50]. Very recently, PI3K/AKT pathway in association with IGF2BP2 has been shown to promote vasculogenic mimicry formation, an alternative process to traditional angiogenesis [51]. Interestingly, vasculogenic mimicry is associated with poor prognosis and high tumor metastasis [52]. Indeed, beside its expression in primary CRC tissue, *IGF2BP2* was also highly expressed in metastatic CRC tissue.

To our knowledge, this is the first study showing a correlation between *IGF2BP2* expression and in vivo tumor growth in PDXs. Further, this study adds functional relevance to the observed correlation in PDO and PDX samples. It has been demonstrated that IGF2BP2 is nearly always the most abundant *IGF2BP* paralogue in human cancers from the TCGA database [44] and was the only *IGF2BP* member whose expression correlated with late stage tumor growth in the current study. Interestingly, also resistance to an array of anticancer drugs could be correlated to *IGF2BP2* expression. IGF2BP2 has been shown to increase resistance of cancer cells against different drugs in different tumor types [33, 53, 54]. The current study also shows that IGF2BP2 may be able to increase resistance toward classical chemotherapeutics as well as targeted therapies, such as tyrosine kinase inhibitors (TKIs). Due to the independence of IGF2BP2 expression from *KRAS/BRAF* mutational status, IGF2BP2 may help to improve therapy decision in the future.

Metabolic reprogramming constitutes one hallmark of cancer [55]. Tumor cells thereby meet their increased energy requirements compared with normal healthy cells. The classical view proposed by Warburg [56], is that cancer cells primarily use glycolysis to meet these bioenergetic needs. However, glycolysis is not a major energy source in all cancer cells [57], and some cancer cells may alternate between OXPHOS and glycolysis. CLIP data suggested binding to RNAs associated with mitochondrial respiration and IGF2BP2 was demonstrated to localize complex I RNA targets to mitochondria in glioblastoma cells [28]. Accordingly, *NDUFAF4*, *CYB5B*, and *COX16* were decreased in *IGF2BP2* KO HCT116 cells. The induction of NDUFA11 upon *IGF2BP2* knockout might be a regulatory feedback mechanism. Recently, HSCs of *Igf2bp2* knockout mice were shown to recapitulate an aged phenotype at least in part by alterations of gene expression related to mitochondrial metabolism [58]. These effects seem to also play a role in sarcomere function in cardiomyocytes [29].

In conclusion, IGF2BP2 promotes tumor growth in vivo and can trigger chemoresistance in CRC potentially by altering the metabolism of the mitochondrial respiratory chain.

## Supplementary Information

**Additional file 1. **Supplementary Methods.

**Additional file 2: Supplementary Figures. Figure S1.** Representative Western blot analysis as validation for the HCT116 *IGF2BP2* knockout (KO) cell line. **Figure S2.** *IGF2BP2* expression is associated with chemoresistance in PDO. **Figure S3.** Comparison of low and high *IGF2BP2* expressing patient derived xenografts (PDX) regarding tumor growth under chemotherapeutic treatment. **Figure S4.** Chemoresistance of HCT116 *IGF2BP2* wildtype (WT) and knockout (KO) cells in 2D cell culture. **Figure S5.** Chemoresistance of HCT116 *IGF2BP2* wildtype (WT) and knockout (KO) cells in 2D cell culture. **Figure S6.** Chemoresistance of HCT116 *IGF2BP2* wildtype (WT) and knockout (KO) cells in 3D cell culture.

**Additional file 3: Supplementary Table S1. Primer sequences. **

**Additional file 4: Supplementary Table S2.** Comparison of the expression of the three IGF2BPs.

**Additional file 5: Supplementary Table S3. gProfiler_hsapiensprimaryTargets**

**Additional file 6: Supplementary Table S4. gProfiler_metastaseTargets**

**Additional file 7: Supplementary Table S5. gProfiler_IntersectionBoth**

## Data Availability

As described in Schütte et al. [31] the complete set of NGS data for patient tumors, xenografts, and cell models are available upon request in the European Genome-phenome Archive (EGA) of the EBI data repository under Accession number EGAS00001001752.

## References

[1] Xi Y, Xu P (2021). Global colorectal cancer burden in 2020 and projections to 2040. Transl Oncol.

[2] Siegel R, Miller K, Sauer A (2020). Colorectal cancer statistics, 2020. Ca-a Cancer Journal For Clinicians.

[3] Vaghari-Tabari M, Majidinia M, Moein S, et al. MicroRNAs and colorectal cancer chemoresistance: New solution for old problem. Life Sci. 2020;259. 10.1016/j.lfs.2020.118255 10.1016/j.lfs.2020.118255 32818543

[4] Schutte M, Risch T, Abdavi-Azar N, et al. Molecular dissection of colorectal cancer in pre-clinical models identifies biomarkers predicting sensitivity to EGFR inhibitors. Nat Commun. 2017;8. 10.1038/ncomms14262 10.1038/ncomms14262 PMC5309787 28186126

[5] Vodenkova S, Buchler T, Cervena K, et al. 5-fluorouracil and other fluoropyrimidines in colorectal cancer: Past, present and future. Pharm Ther. 2020;206. 10.1016/j.pharmthera.2019.107447 10.1016/j.pharmthera.2019.107447 31756363

[6] Kukcinaviciute E, Jonusiene V, Sasnauskiene A (2018). Significance of Notch and Wnt signaling for chemoresistance of colorectal cancer cells HCT116. J Cell Biochem.

[7] Douillard J-Y, Oliner KS, Siena S (2013). Panitumumab–FOLFOX4 Treatment and RAS Mutations in Colorectal Cancer. N Engl J Med.

[8] Van Cutsem E, Köhne CH, Láng I (2011). Cetuximab plus irinotecan, fluorouracil, and leucovorin as first-line treatment for metastatic colorectal cancer: updated analysis of overall survival according to tumor KRAS and BRAF mutation status. J Clin Oncol.

[9] Wang J, Chen L, Qiang P (2021). The role of IGF2BP2, an m6A reader gene, in human metabolic diseases and cancers. Cancer Cell Int.

[10] Samanta S, Pursell B, Mercurio A (2013). IMP3 Protein Promotes Chemoresistance in Breast Cancer Cells by Regulating Breast Cancer Resistance Protein (ABCG2) Expression. J Biol Chem.

[11] Hsu K, Shen M, Huang Y (2015). Overexpression of the RNA-binding proteins Lin28B and IGF2BP3 (IMP3) is associated with chemoresistance and poor disease outcome in ovarian cancer. Br J Cancer.

[12] Boyerinas B, Park S, Murmann A (2012). Let-7 modulates acquired resistance of ovarian cancer to Taxanes via IMP-1-mediated stabilization of multidrug resistance 1. Int J Cancer.

[13] Faye M, Beug S, Graber T (2015). IGF2BP1 controls cell death and drug resistance in rhabdomyosarcomas by regulating translation of cIAP1. Oncogene.

[14] Kim T, Havighurst T, Kim K (2018). Targeting insulin-like growth factor 2 mRNA-binding protein 1 (IGF2BP1) in metastatic melanoma to increase efficacy of BRAF(V600E) inhibitors. Mol Carcinog.

[15] Kessler S, Pokorny J, Zimmer V (2013). IGF2 mRNA binding protein p62/IMP2–2 in hepatocellular carcinoma: antiapoptotic action is independent of IGF2/PI3K signaling. Am J Physiol Gastrointestinal Liver Physiol..

[16] Kessler SM, Laggai S, Barghash A (2015). IMP2/p62 induces genomic instability and an aggressive hepatocellular carcinoma phenotype. Cell Death Dis.

[17] Dahlem C, Abuhaliema A, Kessler SM (2022). First Small-Molecule Inhibitors Targeting the RNA-Binding Protein IGF2BP2/IMP2 for Cancer Therapy. ACS Chem Biol.

[18] Ye S, Song W, Xu X (2016). IGF2BP2 promotes colorectal cancer cell proliferation and survival through interfering with RAF-1 degradation by miR-195. FEBS Lett.

[19] Kessler S, Laggai S, Barghash A, et al. IMP2/p62 induces genomic instability and an aggressive hepatocellular carcinoma phenotype. Cell Death Dis. 2015;6. 10.1038/cddis.2015.241 10.1038/cddis.2015.241 PMC4632283 26426686

[20] Kessler S, Lederer E, Reihs R (2013). Overexpression of the insulin-like growth factor 2 (IGF2) mRNA binding protein (IMP) p62 as a marker of poor outcome in gallbladder cancer. Hepatology.

[21] Dahlem C, Barghash A, Puchas P, et al. The Insulin-Like Growth Factor 2 mRNA Binding Protein IMP2/IGF2BP2 is Overexpressed and Correlates with Poor Survival in Pancreatic Cancer. Int J Mol Sci. 2019;20(13). 10.3390/ijms20133204 10.3390/ijms20133204 PMC6651604 31261900

[22] Kessler S, Lederer E, Laggai S (2017). IMP2/IGF2BP2 expression, but not IMP1 and IMP3, predicts poor outcome in patients and high tumor growth rate in xenograft models of gallbladder cancer. Oncotarget.

[23] Degrauwe N, Schlumpf T, Janiszewska M (2016). The RNA Binding Protein IMP2 Preserves Glioblastoma Stem Cells by Preventing let-7 Target Gene Silencing. Cell Rep.

[24] Janiszewska M, Suva M, Riggi N (2012). Imp2 controls oxidative phosphorylation and is crucial for preserving glioblastoma cancer stem cells. Genes Dev.

[25] Chao W, D'Amore PA (2008). IGF2: Epigenetic regulation and role in development and disease. Cytokine Growth Factor Rev.

[26] Tybl E, Shi F-D, Kessler SM (2011). Overexpression of the IGF2-mRNA binding protein p62 in transgenic mice induces a steatotic phenotype. J Hepatol.

[27] Kessler SM, Haybaeck J, Kiemer AK (2016). Insulin-Like Growth Factor 2 - The Oncogene and its Accomplices. Curr Pharm Des.

[28] Janiszewska M, Suvà ML, Riggi N (2012). Imp2 controls oxidative phosphorylation and is crucial for preservin glioblastoma cancer stem cells. Genes Dev.

[29] Ladha FA, Thakar K, Pettinato AM (2021). Actinin BioID reveals sarcomere crosstalk with oxidative metabolism through interactions with IGF2BP2. Cell Reports..

[30] Krumbein M, Oberman F, Cinnamon Y, et al. IGF2BP2 is Induced by Stress in the Heart and Mediates Dilated Cardiomyopathy. bioRxiv 2022:2022.11.03.515033. 10.1101/2022.11.03.515033

[31] Schütte M, Risch T, Abdavi-Azar N (2017). Molecular dissection of colorectal cancer in pre-clinical models identifies biomarkers predicting sensitivity to EGFR inhibitors. Nat Commun.

[32] Golob-Schwarzl N, Schweiger C, Koller C (2017). Separation of low and high grade colon and rectum carcinoma by eukaryotic translation initiation factors 1, 5 and 6. Oncotarget.

[33] Kessler SM, Pokorny J, Zimmer V (2013). IGF2 mRNA binding protein p62/IMP2-2 in hepatocellular carcinoma: antiapoptotic action is independent of IGF2/PI3K signaling. Am J Physiol Gastrointest Liver Physiol.

[34] Lu M, Nakamura RM, Dent ED (2001). Aberrant Expression of Fetal RNA-Binding Protein p62 in Liver Cancer and Liver Cirrhosis. Am J Pathol.

[35] Barghash AG-S, N, Helms V, Haybaeck J, Kessler SM (2016). Elevated expression of the IGF2 mRNA binding protein 2 (IGF2BP2/IMP2) is linked to short survival and metastasis in esophageal adenocarcinoma. Oncotarget.

[36] Kessler SM, Lederer E, Laggai S (2017). IMP2/IGF2BP2 expression, but not IMP1 and IMP3, predicts poor outcome in patients and high tumor growth rate in xenograft models of gallbladder cancer. Oncotarget.

[37] Erdmann F, Prell E, Jahreis G (2018). Screening for Selective Protein Inhibitors by Using the IANUS Peptide Array. ChemBioChem.

[38] Consortium EP (2012). An integrated encyclopedia of DNA elements in the human genome. Nature.

[39] Dehghani Amirabad A, Ramasamy P, Wierz M (2018). Transgenic expression of the RNA binding protein IMP2 stabilizes miRNA targets in murine microsteatosis. Biochimica et Biophysica Acta (BBA) - Mol Basis Dis..

[40] Benjamini Y, Hochberg Y (1995). Controlling the False Discovery Rate: A Practical and Powerful Approach to Multiple Testing. J Roy Stat Soc: Ser B (Methodol).

[41] Raudvere U, Kolberg L, Kuzmin I (2019). g:Profiler: a web server for functional enrichment analysis and conversions of gene lists (2019 update). Nucleic Acids Res.

[42] Vandesompele J, De Preter K, Pattyn F (2002). Accurate normalization of real-time quantitative RT-PCR data by geometric averaging of multiple internal control genes. Genome Biol.

[43] Liu R, Chen Y, Liu G (2020). PI3K/AKT pathway as a key link modulates the multidrug resistance of cancers. Cell Death Dis.

[44] Dai N, Ji F, Wright J, et al. IGF2 mRNA binding protein-2 is a tumor promoter that drives cancer proliferation through its client mRNAs IGF2 and HMGA1. Elife 2017;6. 10.7554/eLife.27155 10.7554/eLife.27155 PMC5576481 28753127

[45] Hu X, Peng W-X, Zhou H (2020). IGF2BP2 regulates DANCR by serving as an N6-methyladenosine reader. Cell Death Differ.

[46] Li T, Hu P-S, Zuo Z (2019). METTL3 facilitates tumor progression via an m6A-IGF2BP2-dependent mechanism in colorectal carcinoma. Mol Cancer.

[47] Ahn S-M, Jang SJ, Shim JH, et al. Genomic portrait of resectable hepatocellular carcinomas: Implications of RB1 and FGF19 aberrations for patient stratification. Hepatology 2014;in press:n/a. 10.1002/hep.27198 24798001

[48] Liu W, Li Z, Xu W (2013). Humoral autoimmune response to IGF2 mRNA-Binding protein (IMP2/p62) and its tissue-specific expression in colon cancer. Scand J Immunol.

[49] Cui J, Tian J, Wang W (2021). IGF2BP2 promotes the progression of colorectal cancer through a YAP-dependent mechanism. Cancer Sci.

[50] Dai N, Christiansen J, Nielsen FC (2013). mTOR complex 2 phosphorylates IMP1 cotranslationally to promote IGF2 production and the proliferation of mouse embryonic fibroblasts. Genes Dev.

[51] Liu X, He H, Zhang F (2022). m6A methylated EphA2 and VEGFA through IGF2BP2/3 regulation promotes vasculogenic mimicry in colorectal cancer via PI3K/AKT and ERK1/2 signaling. Cell Death Dis.

[52] Zhang X, Zhang J, Zhou H (2019). Molecular Mechanisms and Anticancer Therapeutic Strategies in Vasculogenic Mimicry. J Cancer.

[53] Han J, Yu X, Wang S (2021). IGF2BP2 Induces U251 Glioblastoma Cell Chemoresistance by Inhibiting FOXO1-Mediated PID1 Expression Through Stabilizing lncRNA DANCR. Front Cell Dev Biol..

[54] Sa R, Liang R, Qiu X (2022). IGF2BP2-dependent activation of ERBB2 signaling contributes to acquired resistance to tyrosine kinase inhibitor in differentiation therapy of radioiodine-refractory papillary thyroid cancer. Cancer Lett.

[55] Hanahan D, Weinberg RA (2011). Hallmarks of cancer: The next generation. Cell.

[56] Heiden MGV, Cantley LC, Thompson CB (2009). Understanding the Warburg Effect: The Metabolic Requirements of Cell Proliferation. Science.

[57] Jose C, Bellance N, Rossignol R (2011). Choosing between glycolysis and oxidative phosphorylation: A tumor's dilemma?. Biochimica et Biophysica Acta (BBA) - Bioenergetics..

[58] Suo M, Rommelfanger MK, Chen Y (2022). Age-dependent effects of Igf2bp2 on gene regulation, function, and aging of hematopoietic stem cells in mice. Blood.

