# Supplementary material for: A combined computational and functional approach identifies IGF2BP2 as a driver of chemoresistance in a wide array of pre-clinical models of colorectal cancer
Source: Mol Cancer. 2023 May 30;22:89. doi: 10.1186/s12943-023-01787-x (PMC10227963; doi:10.1186/s12943-023-01787-x)
Supplement: Supplementary file 1 — Additional file 1. Supplementary Methods. [file 12943_2023_1787_MOESM1_ESM.pdf]

A combined computational and functional approach identifies IGF2BP2 as a driver of chemoresistance in a wide array of pre-clinical models of colorectal cancer

Sandra Kendzia[1], Susanne Franke[1], Tarek Kröhler[2], Nicole Golob-Schwarzl[3,4,5], Caroline Schweiger[3,6], Anna M. Toeglhofer[3], Christina Skofler[3,4], Stefan Uranitsch[7], Amin El-Heliebi[4,8,9], Julia Fuchs[8,10], Andreas Punschart[11], Philipp Stiegler[11], Marlen Keil[12], Jens Hoffmann[12], David Henderson[13], Hans Lehrach[14], Marie-Laure Yaspo[14], Christoph Reinhard[15,16], Reinhold Schäfer[6], Ulrich Keilholz[6], Christian Regenbrecht[16,17], Rudolf Schicho[18], Peter Fickert[19], Sigurd F. Lax[20], Frank Erdmann[1], Marcel H. Schulz[21], Alexandra K. Kiemer[2], Johannes Haybaeck[3,22], Sonja M. Kessler*[1,2,3,23]

[1] Institute of Pharmacy, Experimental Pharmacology for Natural Sciences, Martin Luther University Halle-Wittenberg, 06120 Halle, Germany

[2] Department of Pharmacy, Pharmaceutical Biology, Saarland University, Saarbrücken, Germany

[3] Diagnostic & Research Center for Molecular Biomedicine, Institute of Pathology, Medical University of Graz, Graz, Austria

[4] Center for Biomarker Research in Medicine (CBmed), Graz, Austria

[5] Department of Dermatology and Venereology, Medical University of Graz, Austria

[6] Charité Comprehensive Cancer Center, Charité - Universitätsmedizin Berlin, 10117 Berlin, Germany

[7] Department of Surgery, Hospital Brothers of Charity Graz, Austria

[8] Division of Cell Biology, Histology and Embryology, Gottfried Schatz Research Center, Medical University of Graz, Austria

[9] BioTechMed-Graz, Graz, Austria

[10] Division of Medical Physics and Biophysics, Medical University Graz, Austria

[11] Department of Surgery, Medical University of Graz, Austria

[12] Experimental Pharmacology & Oncology Berlin GmbH-Berlin-Buch, Germany

[13] Bayer AG, Berlin, Germany

[14] Max Planck Institute for Molecular Genetics, Berlin, Germany

[15] Eli Lilly & Company, Indianapolis, USA

[16] CELLphenomics GmbH, Germany

[17] Institute for Pathology, University Hospital Göttingen, Göttingen, Germany

[18] Division of Pharmacology, Medical University of Graz, Austria

[19] Division of Gastroenterology and Hepatology, Medical University of Graz, Austria

[20] Department of Pathology, Hospital Graz South-West and School of Medicine, Johannes Kepler University Linz, Austria

[21] Institute for Cardiovascular Regeneration, Goethe-University Hospital, 60590 Frankfurt, Germany

[22] Institute of Pathology, Neuropathology and Molecular Pathology, Medical University of Innsbruck, Innsbruck, Austria

[23] Halle Research Centre for Drug Therapy (HRCDT)

Supplementary method

Western blot

Cells were lysed in lysis buffer (50 mm Tris-HCl, 1% (m/v) SDS, 10% (v/v) glycerol, 5% (v/v) 2-mercaptoethanol, 0.004% (m/v) bromphenol blue) supplemented with 1 mM sodiumorthovanadate, 1 mM PMSF and a protease inhibitor mixture (Complete®, Roche Diagnostics, Mannheim, Germany) according to the manufacturer's instructions. SDS-polyacrylamide gel electrophoresis (SDS-PAGE) was done using the Bio-Rad Mini PROTEAN system (Bio-Rad, München, Germany). The Mini-Transblot cell (Bio-Rad, München, Germany) system was employed to transfer separated protein samples onto a polyvinylidene fluoride (PVDF) membrane (Immobilon-FL, Millipore, Schwalbach am Taunus, Germany). Membrane was blocked using Rockland Blocking Buffer (#MB-070, Rockland Immunochemicals). Antibodies used were specific for IMP2/p62 [1] and b-actin (#D6A8, Cell Signaling). Both primary antibodies were diluted 1:1000 in Rockland Blocking Buffer and incubated for 1h at room temperature. Secondary HRP-conjugated antibody (Dianova) was diluted 1:5000 in 5% milk powder in TBS-T and incubated for 1 h inkubation at room temperature. For detection ECL Prime Western Blotting Detection Reagent (GE Healthcare Amersham) was used according to manufacturer’s guidelines. Detection was performed with the Octoplus QPLEX Analyzer (nhDiagnostics, Halle, Germany).

1 Lu M, Nakamura RM, Dent ED, et al. Aberrant Expression of Fetal RNA-Binding Protein p62 in Liver Cancer and Liver Cirrhosis. *Am J Pathol* 2001;159(3):945-53.
