# Supplementary material for: A combined computational and functional approach identifies IGF2BP2 as a driver of chemoresistance in a wide array of pre-clinical models of colorectal cancer
Source: Mol Cancer. 2023 May 30;22:89. doi: 10.1186/s12943-023-01787-x (PMC10227963; doi:10.1186/s12943-023-01787-x)
Supplement: Supplementary file 2 — Additional file 2: Supplementary Figures. Figure S1. Representative Western blot analysis as validation for the HCT116 IGF2BP2 knockout (KO) cell line. Figure S2. IGF2BP2 expression is associated with chemoresistance in PDO. Figure S3. Comparison of low and high IGF2BP2 expressing patient derived xenografts (PDX) regarding tumor growth under chemotherapeutic treatment. Figure S4. Chemoresistance of HCT116 IGF2BP2 wildtype (WT) and knockout (KO) cells in 2D cell culture. Figure S5. Chemoresistance of HCT116 IGF2BP2 wildtype (WT) and knockout (KO) cells in 2D cell culture. Figure S6. Chemoresistance of HCT116 IGF2BP2 wildtype (WT) and knockout (KO) cells in 3D cell culture. [file 12943_2023_1787_MOESM2_ESM.pdf]

Supplementary Figure S1_Validation of HCT116 IGF2BP2 KO

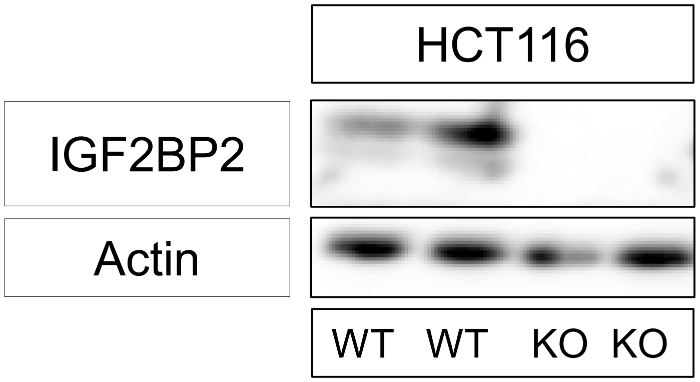

Figure S1: Representative Western blot analysis as validation for the HCT116 IGF2BP2 knockout (KO) cell line.

Western blot for IGF2BP2 in HCT116 IGF2BP2 knockout cells in comparison to HCT116 wildtype (WT) cells. Beta-actin was used as loading control.

Supplementary Figure S2_Data correlation analysis

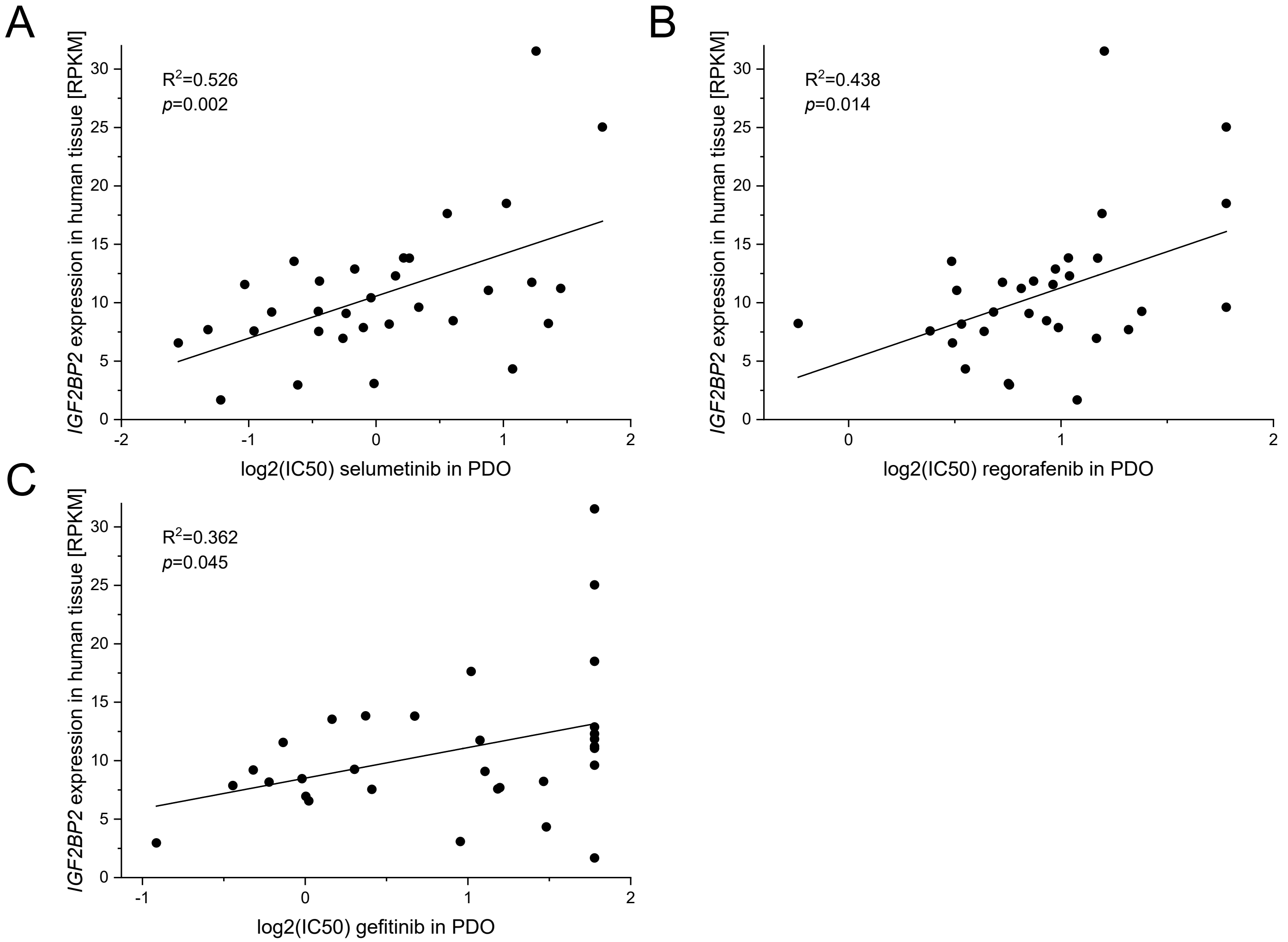

Figure S2: *IGF2BP2* expression is associated with chemoresistance in PDO.

A-C: Graphs show significant Pearson correlation coefficients (R^2) and respective *p*-values for correlation analysis of *IGF2BP2* expression in primary tumor tissues with logIC50 values of the respective drug in PDOs. LogIC50 values have previously been published in Supplementary Data 14 in Schütte et al.

Supplementary Figure S3_Comparison of low and high *IGF2BP2* expressing PDX regarding tumor growth under chemotherapeutic treatment.

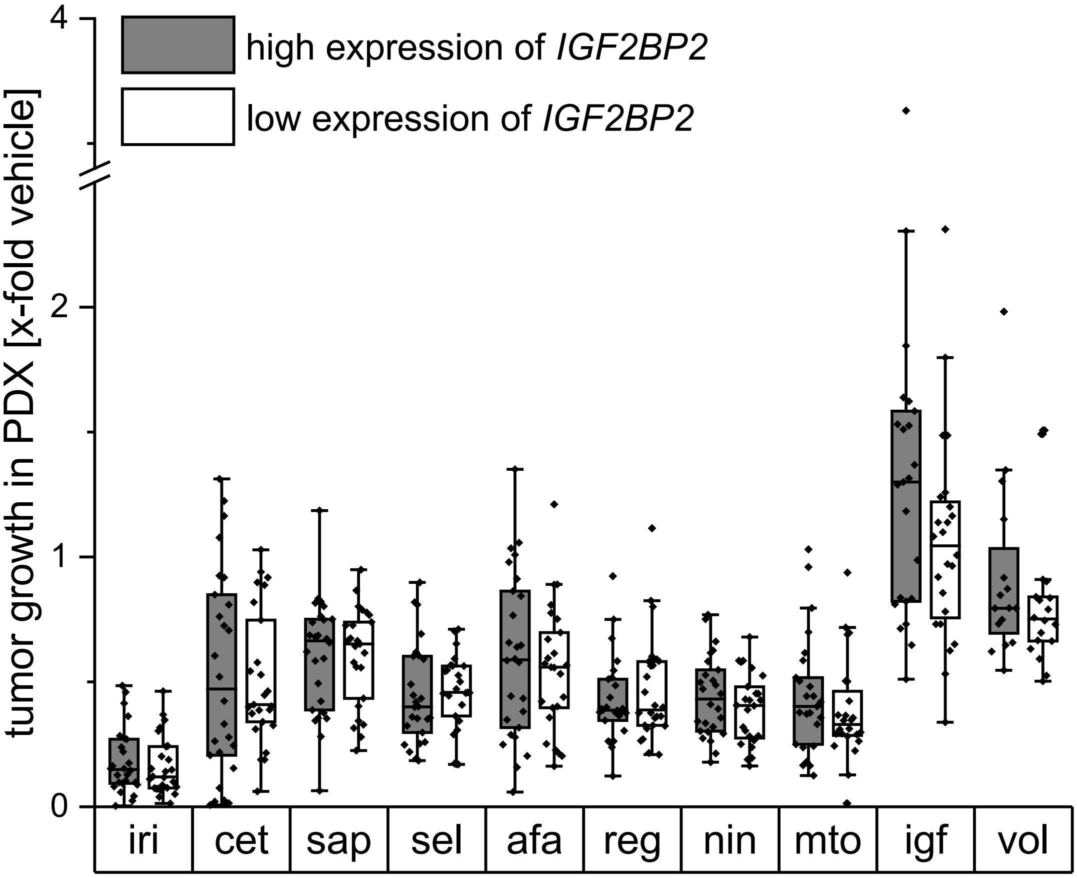

Figure S3: Comparison of low and high *IGF2BP2* expressing patient derived xenografts (PDX) regarding tumor growth under chemotherapeutic treatment.

PDX samples of primary tumor and liver metastasis tissue (n=52) were divided in high (above median) and low (below median) *IGF2BP2* expressing groups. PDX tumor growth under treatment with the respective drug was plotted for high and low *IGF2BP2* expressing samples. The used drugs were irinotecan (iri), cetuximab (cet), sapitinib (sap), selumitinib (sel), afatinib (afa), regorafenib (reg), nintedanib (nin), BI860585 (mto), BI836842 (igf) and voltinib (vol). Statistical significance was determined by Mann-Whitney U Test for not normally distributed samples or Student´s t-test for normally distributed samples.

Supplementary Figure S4_2D data_IC50

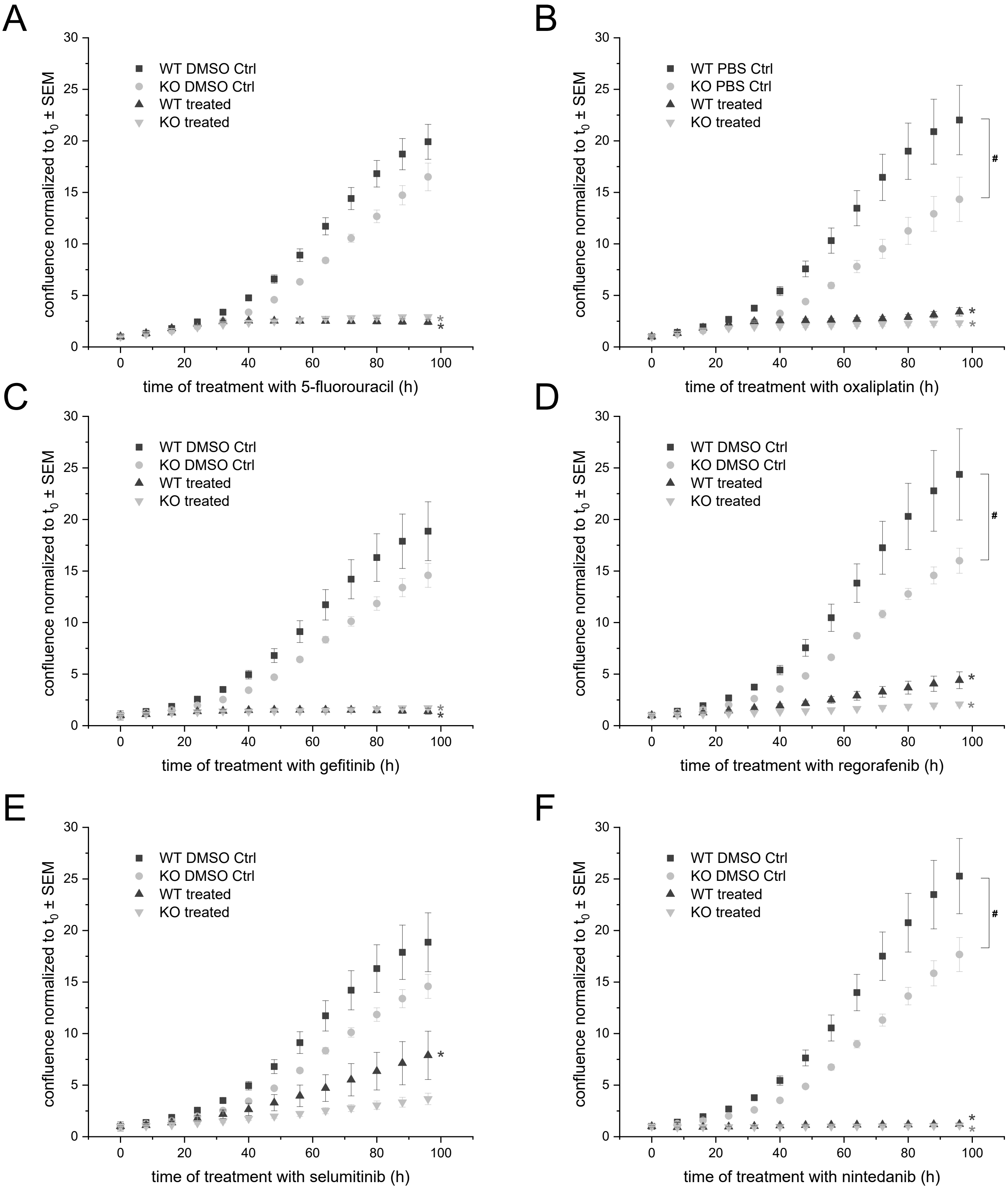

Figure S4: Chemoresistance of HCT116 IGF2BP2 wildtype (WT) and knockout (KO) cells in 2D cell culture.
A-F: Cell confluency was monitored using the IncuCyte®S3 system over 4 days. HCT116 WT cells and HCT116 IGF2BP2 KO cells were seeded 24h prior treatment. Cells were treated with IC50 concentrations of 5-fluorouracil (A, 22.2 µM), oxaliplatin (B, 2.6 µM), gefitinib (C, 23.5 µM), regorafenib (D, 8.1 µM), selumitinib (E, 7.4 µM), nintedanib (F, 8.2 µM) and the vehicle control (Ctrl). Confluency was normalized to the time of treatment (0h). Data are represented as means ±SEM, n=3 (quadruplicates). Statistical analysis was performed with a two-way ANOVA using the area under the curve. Asterisks represent *p* values for the comparisons between the untreated and treated condition in the respective cell line. Hashmarks stand for *p* values labeling differences in growth between both cell lines either control or treated condition (*p* values were $p \leq 0.05$).

Supplementary Figure S5_2D data_IC20

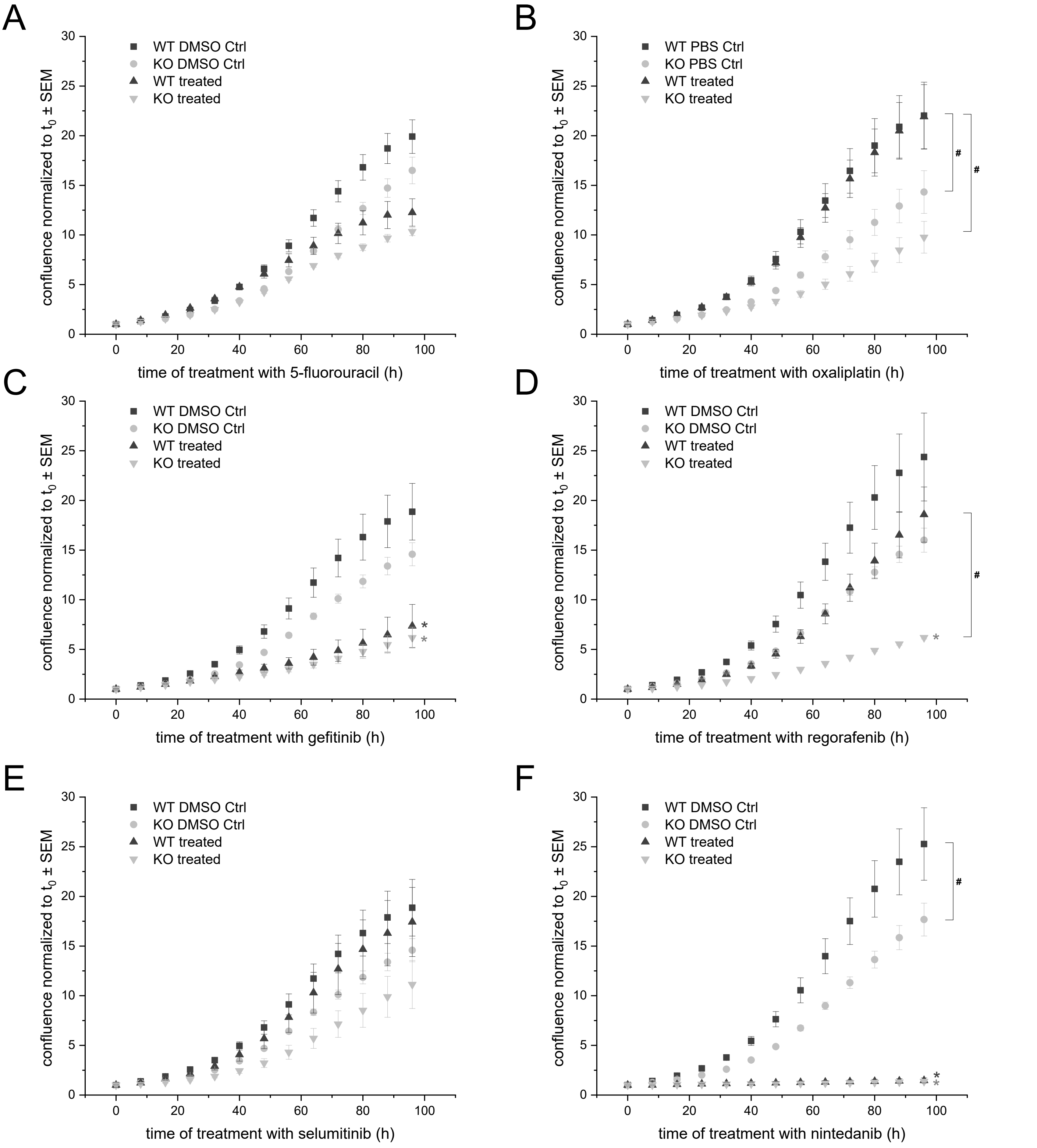

Figure S5: Chemoresistance of HCT116 IGF2BP2 wildtype (WT) and knockout (KO) cells in 2D cell culture.
A-F: Cell confluency was monitored using the IncuCyte®S3 system over 4 days. HCT116 WT cells and HCT116 IGF2BP2 KO cells were seeded 24h prior treatment. Cells were treated with IC20 concentrations of 5-fluorouracil (A, 3.8 µM), oxaliplatin (B, 0.3 µM), gefitinib (C, 15.5 µM), regorafenib (D, 3.2 µM), selumitinib (E, 0.01 µM), nintedanib (F, 6.6 µM) and the vehicle control (Ctrl). Confluency was normalized to the time of treatment (0h). Data are represented as means ±SEM, n=3 (quadruplicates). Statistical analysis was performed with a two-way ANOVA using the area under the curve. Asterisks represent *p* values for the comparisons between the untreated and treated condition in the respective cell line. Hashmarks stand for *p* values labeling differences in growth between both cell lines either control or treated condition (*p* values were $p \leq 0.05$).

Supplementary Figure S6_3D data_IC20

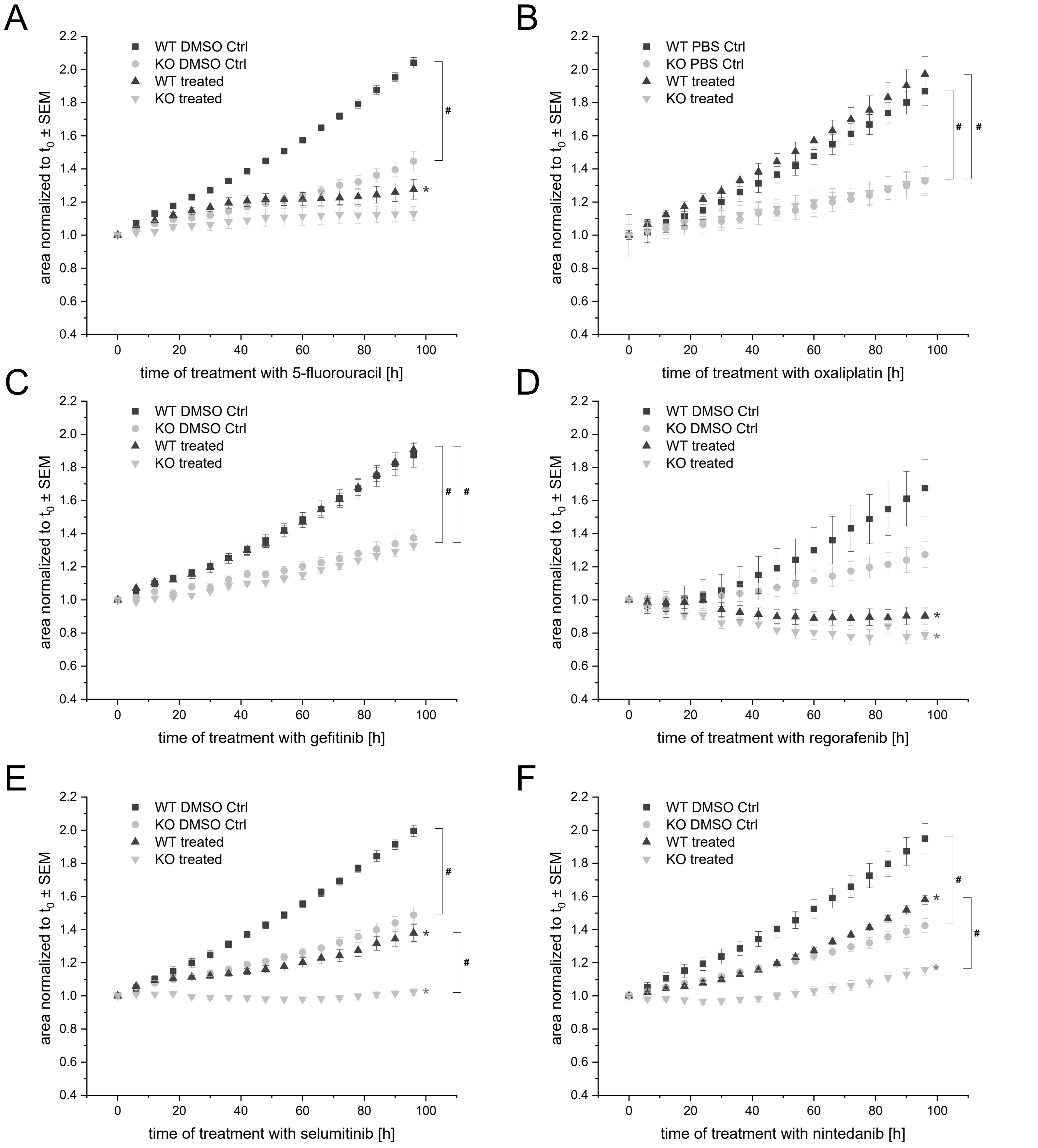

Figure S6: Chemoresistance of HCT116 IGF2BP2 wildtype (WT) and knockout (KO) cells in 3D cell culture. A-F: Spheroid growth of HCT116 WT and HCT116 IGF2BP2 KO cells was monitored for 96h by automated live-cell microscopy, starting after spheroid formation. Cells were treated with IC20 concentrations of 5-fluorouracil (A, 3.8 µM), oxaliplatin (B, 0.3 µM), gefitinib (C, 15.5 µM), regorafenib (D, 3.2 µM), selumitinib (E, 0.01 µM), nintedanib (F, 6.6 µM) and the vehicle control (Ctrl). Spheroid area was analyzed using the IncuCyte®S3 system and was normalized to 3-day old spheroids. Data are represented as means ± SEM, n=3 (quadruplicates). Statistical analysis was performed with a two-way ANOVA using the area under the curve. Asterisks represent *p* values for the comparisons between the untreated and treated condition in the respective cell line. Hashmarks stand for *p* values labeling differences in growth between both cell lines either control or treated condition (*p* values were $p \leq 0.05$).
